# Comprehensive phytochemical profiling of dealcoholized zhuyeqing liquor extract and its protective effect on hyperuricemia by regulating urate transporters and LPS/NLRP3 inflammasome pathway

**DOI:** 10.3389/fnut.2026.1876752

**Published:** 2026-07-29

**Authors:** Bingye Xu, Xiaoxiao Li, Rong Liu, Ying Han, Pan Zhen, Hanyue Fu, Huan Lv, Fan Wei, Haoxin Cui, Bowei Zhang, Shuo Wang

**Affiliations:** 1 Tianjin Key Laboratory of Food Science and Health, School of Medicine, Nankai University, Tianjin, China; 2 Xinghuacun Fenjiu Distillery Co., Ltd., Fenyang, Shanxi, China

**Keywords:** anti-inflammatory, dealcoholized zhuyeqing liquor extract (ZLE), hyperuricemia (HUA), medicine and food homology (MFH), NLRP3 inflammasome, renoprotection, urate transporters, xanthine oxidase (XOD)

## Abstract

**Introduction:**

Hyperuricemia (HUA) is a major risk factor for gout and renal injury, underscoring the need for safe and effective natural dietary interventions. This study investigated the chemical composition and antihyperuricemic effects of dealcoholized Zhuyeqing liquor extract (ZLE) using both cellular and animal models.

**Methods:**

The nutritional composition and phytochemical profile of ZLE were characterized by quantitative analysis and ultra-performance liquid chromatography–tandem mass spectrometry (UPLC–MS/MS). The antihyperuricemic activity of ZLE was evaluated in adenosine-induced HepG2 cells and in a hypoxanthine/potassium oxonate-induced mouse model of HUA by assessing uric acid (UA) metabolism, hepatorenal function, urate transporter expression, and renal inflammatory signaling.

**Results:**

Quantitative analysis showed that ZLE is rich in polysaccharides, terpenes, and flavonoids. UPLC–MS/MS identified 389 phytochemical constituents, predominantly iridoid glycosides, organic acids, and flavonoids. In adenosine-induced HepG2 cells, ZLE significantly reduced UA production and xanthine oxidase (XOD) activity. In HUA mice, ZLE dose-dependently decreased serum UA levels, improved renal and hepatic function by reducing serum creatinine, blood urea nitrogen, alanine aminotransferase, and aspartate aminotransferase levels, and inhibited hepatic XOD activity. Furthermore, ZLE restored renal urate homeostasis through coordinated regulation of key urate transporters and alleviated HUA-induced renal inflammation and histopathological injury by suppressing the lipopolysaccharide-mediated NLRP3/Caspase-1 signaling pathway.

**Discussion:**

These findings demonstrate that ZLE exerts antihyperuricemic and renoprotective effects through coordinated modulation of uric acid production, renal urate transport, and inflammatory signaling. The results support the potential application of ZLE as a functional food ingredient for dietary management of HUA and provide a scientific basis for translating traditional dietary resources into modern nutritional interventions.

## 1 Introduction

Hyperuricemia (HUA) is defined as a metabolic imbalance characterized by abnormally high levels of uric acid (UA) in the bloodstream. This condition arises when circulating urate concentrations exceed the body’s physiological homeostatic range. Typically, HUA results from either an overproduction of UA or an impaired ability of the kidneys to eliminate it efficiently (1). In addition to gout, HUA is also a predisposing factor for chronic kidney disease, hypertension, and cardiovascular disease (2). Hepatic tissue plays a central role in purine catabolism. Within this process, xanthine oxidase (XOD) acts as a key enzyme. It functions by catalyzing the conversion of hypoxanthine (HX) and xanthine into urate, the final metabolite (3). Consequently, XOD inhibitors like allopurinol and febuxostat are clinical mainstays; However, their long-term use is often limited by side effects, including hypersensitivity (4) and cardiovascular toxicity (5). Moreover, considering that renal underexcretion accounts for approximately 90% of primary HUA cases, targeting renal excretion pathways is equally critical (6).

The kidney handles approximately 70% of daily urate excretion through a complex system of transporters located in the proximal renal tubule (6). This process relies on a delicate balance between reabsorption and secretion. Urate anion transporter 1 (URAT1) and glucose transporter type 9 (GLUT9) are the distinct “gatekeepers” responsible for reabsorbing urate back into the systemic circulation, while organic anion transporter 1 (OAT1) and ATP-binding cassette subfamily G member 2 (ABCG2) promote urate secretion into the urine (7). Dysregulation of these transporters is a significant cause of HUA (8). Recent studies also emphasize that HUA-induced renal injury is frequently worsened by systemic stressors, such as the translocation of gut-derived lipopolysaccharide (LPS), which triggers the activation of the NOD-like receptor thermal protein domain associated protein 3 (NLRP3) inflammasome and subsequent inflammatory cascades within the renal parenchyma (9, 10).

Zhuyeqing liquor (ZYQ) is a renowned traditional Chinese functional beverage with origins dating to the Warring States era. It gained wide popularity among the public during the Southern and Northern Dynasties. With the rapid advancement of society, ZYQ flourished in the Tang Dynasty and Song Dynasty, and was officially approved as a functional beverage by the Ministry of Public Health of China in 1998. ZYQ is formulated based on the “herbal health-preserving” theory of the *Compendium of Materia Medica*. It is crafted from twelve authentic medicinal herbs, including eight medicine and food homology (MFH) substances: *Lophatherum gracile* Brongn. (Zhuye), *Gardenia jasminoides* Ellis (Zhizi), *Angelica sinensis* (Oliv.) Diels (Danggui), *Kaempferia galanga* L. (Shannai), *Citrus reticulata* Blanco (Chenpi), *Chrysanthemum morifolium* Ramat. (Juhua), *Amomum villosum* Lour. (Sharen), *Eugenia caryophyllata* Thunb. (Dingxiang), alongside four rare botanicals including *Santalum album* L. (Tanxiang), *Lysimachia foenum-graecum* Hance (Linglingxiang), *Aucklandia lappa* Decne. (Muxiang), *Lysimachia capillipes* Hemsl. (Paicao) (11). In Traditional Chinese Medicine (TCM), ZYQ is known for its “heat-clearing and dampness-removing” effects, which is consistent with the principle of treating damp-heat syndromes such as gout (12).

Different phytochemical components may mutually enhance their solubility in alcohol and *in vivo* bioavailability. As a classic traditional Chinese medicinal formula, the various medicinal herbs in ZYQ may exert synergistic effects (13, 14). Modern research has begun to reveal the nutritional and bioactive profiles of ZYQ. Phytochemical profiling has identified 61 compounds, such as flavonoids and phenolic acids (15). Functionally, ZYQ has been shown to exert comprehensive protective effects, including anti-inflammatory properties, potent antioxidant (16) and hepatoprotective activities (11, 17), and antidepressant effects (18). Flavonoids in Zhuye exhibit potent antioxidant activity (19). Other herbal components in ZYQ may regulate urate transporters (20) and strengthen the gut barrier (21, 22).

The combined action of ZYQ extract on renal transporter proteins remains unclear (23). How it comprehensively protects kidneys also needs more research. This study aims to fill these gaps. We first conducted a detailed chemical composition analysis of dealcoholized zhuyeqing liquor extract (ZLE) and used ultra-performance liquid chromatography coupled with quadrupole-exactive HF mass spectrometry (UPLC-Q-Exactive HF-MS) to identify phytochemical components. Subsequently, we constructed cell and mouse models to study the specific effects and possible mechanisms of ZLE intervention on UA metabolism. This research seeks to provide a scientific foundation for the application of ZLE as a functional food component in the dietary management of HUA and its related renal complications.

## 2 Materials and methods

### 2.1 Main materials

ZYQ was provided by Shanxi Xinghuacun Fenjiu Distillery Co., Ltd. (Shanxi, China). XOD, inosine and febuxostat were obtained from Shanghai Macklin Biochemical Co., Ltd. (Shanghai, China). UA, adenine, xanthine, adenosine, HX and allopurinol standards were from Aladdin Reagent Co., Ltd. (Shanghai, China). Sodium carboxymethyl cellulose (CMC) was bought from Solarbio Science and Technology Co., Ltd. (Beijing, China). 3-(4,5-Dimethylthiazol-2-yl)-2,5-diphenyltetrazolium bromide (MTT) was bought from Shanghai Yuanye Bio-Technology Co., Ltd. (Shanghai, China). Dimethyl sulfoxide (DMSO) and potassium oxazinate (PO) were purchased from Sigma-Aldrich (St. Louis, MO, United States). Dulbecco’s modified eagle’s medium (DMEM), penicillin streptomycin and fetal bovine serum (FBS) were purchased from Gibco Co., Ltd. (Carlsbad, United States). The assay kits for XOD and LPS were provided by Fankui Technology Co., Ltd. (Shanghai, China). The activity assay kit for Caspase-1 and the ELISA kits for Caspase-1, IL-1*β*, and IL-18 were purchased from Elabscience (Wuhan, China). URAT1, ABCG2 and *β*-actin antibodies were obtained from Wanlei Life Sciences Co., Ltd. (Shenyang, China). Antibodies for GLUT9 were obtained from Proteintech Group, Inc. (Wuhan, China), while that for OAT1 was obtained from Affinity Biosciences (Changzhou, China). Primers for RT-qPCR were combined by TsingKe Biological Technology (Beijing, China).

### 2.2 Preparation of ZLE

The voucher specimen of ZYQ was preserved at Nankai University (Tianjin, China). Its registration number is ZYQ 20240701. ZLE was obtained by rotary evaporation of the liquor at 50 °C to remove ethanol, followed by lyophilization. The yield was 1.97 g/100 mL.

### 2.3 Gas chromatograph-mass spectrometric analysis

To determine the residual ethanol in the freeze-dried ZLE powder, GC-MS measurements were performed using an Agilent 7890B-7000D system (Agilent Technologies Inc., California, United States) with a DB-WAX column (30 m × 0.25 mm × 0.5 μm). Operating conditions were as follows: carrier gas: Nitrogen with a flow rate of 1 mL/min; column temperature program: 8 min at 40 °C, 40–220 °C at 50 °C/min and finally 4 min at 220 °C. The temperature of the injection port was 200 °C. The split ratio was 10:1. The MS was equipped with an electron ionization (EI) source, and the operating conditions were as follows: ion source temperature 250 °C, quadrupole temperature 150 °C. The measurements were carried out in selected ion monitoring (SIM) mode (24).

### 2.4 Macronutrient quantification

Protein and peptides were determined via the Kjeldahl method (25), using 10 kDa molecular weight cut off (MWCO) filters to differentiate fractions. Polysaccharides, total terpenes, and total flavonoids were quantified using the phenol-sulfuric acid (26), vanillin-perchloric acid (27), and aluminum nitrate (28) colorimetric methods, respectively.

### 2.5 UPLC-Q-Exactive HF-MS analysis of ZLE chemical constituents

ZLE (reconstituted in 70% methanol) was analyzed using a Thermo Vanquish ultra-high performance liquid chromatography (UHPLC) coupled with a Q Exactive HF mass spectrometer (29). Separation was achieved on a Zorbax Eclipse C18 column (1.8 μm × 2.1 mm × 100 mm) with a flow rate of 0.3 mL/min. The column temperature was maintained at 30 °C. The mobile phases were 0.1% formic acid in water (A) and acetonitrile (B). The gradient was: 0–2 min, 5% B; 2–6 min, 5–30% B; 6–7 min, 30% B; 7–12 min, 30–78% B; 12–14 min, 78% B; 14–17 min, 78–95% B; 17–20 min, 95% B; 20–21 min, 95–5% B; and 21–25 min, 5% B. The injection volume was 2 μL.

The electrospray ionization (ESI) source was operated in both positive and negative modes with the following settings: spray voltage, 3.5 kV; capillary temperature, 330 °C; heater temperature, 325 °C; S-Lens RF level, 55%; and sheath/auxiliary/sweep gas flow rates, 45/15/1 arb. Data were acquired in Data Dependent Acquisition (DDA) mode (TopN = 5) with a scan range of *m/z* 100–1,500. Resolutions were set at 120,000 for full MS and 60,000 for higher collisional dissociation (HCD)-fragmented MS^2^ scans.

In the absence of authentic standards, compounds were identified by matching their accurate mass (mass error < 5 ppm) and MS/MS fragmentation patterns with those in the Thermo mzCloud (online) and mzVault (local) databases using Compound Discoverer 3.3 (Thermo Fisher Scientific). Detailed identification parameters, including retention times (RT), mass errors (Annot. DeltaMass), chemical classifications (Class), and relative contents (%), are provided in Supplementary Table S1.

### 2.6 Evaluation of ZLE bioactivity *in vitro* model

#### 2.6.1 Cell culture and cell treatment

HepG2 cells were cultured in DMEM supplemented with 10% FBS and 1% penicillin–streptomycin at 37 °C in a 5% CO_2_ atmosphere (30). For bioactivity evaluation, HepG2 cells were seeded in 96-well plates and divided into four groups: Control (NC), Model (MD), Febuxostat (0.02 mmol/L, FB), and ZLE (200, 250 mg/L). Following 24 h of drug/extract pretreatment, the cells (except the NC) were treated with 2.5 mmol/L adenosine for 24 h to induce a hyperuricemic state. Subsequently, XOD (0.005 U/mg) was added for a final 12 h incubation. The concentrations of ZLE (200–250 mg/L) used in subsequent experiments were selected based on the MTT assay results, which demonstrated no significant cytotoxicity within this concentration range.

#### 2.6.2 HPLC and biochemical analysis of supernatants

Concentrations of urate and related purine intermediates (xanthine, HX, inosine, adenosine, and adenine) in culture supernatants were measured using high-performance liquid chromatography (31). Chromatographic conditions: Using a ZORBAX SB-Aq column (4.6 mm × 250 mm, 5 μm) equipped with a photodiode array detector, with detection at 260 nm, column temperature at 35 °C. The mobile phase is a mixture of water solution containing 0.1% trifluoroacetic acid (A) and acetonitrile (B). The elution gradient is as follows: 0–7 min, 98.3% A; 7–14 min, 98.3–97% A; 14–21 min, 97–90% A; 21–28 min, 90–70% A; 28–30 min, 70–98.3% A; 30–38 min, 98.3% A. The flow rate is 1.0 mL/min, and the injection volume is 10 μL. Additionally, XOD activity in the supernatants was measured using a commercial ELISA kit.

### 2.7 Animal experiments designed

#### 2.7.1 Test animals

Specific-pathogen-free (SPF) Kunming male mice aged 5 weeks were purchased from Vital River Laboratory Animal Technology Co., Ltd. (Beijing, China). The mice were raised at the SPF environment with a suitable temperature of (22 ± 2) °C and relative humidity of 50–60%, and alternating 12 h light–dark cycles. All mice were euthanized by isofluorane anesthesia followed by cervical dislocation. Animal experiments were strictly conducted under the guidelines of Animal Care and Use Committee of Nankai University (Permit Number: SYXK (Jin) 2019-0001).

#### 2.7.2 HUA model and drug administration

After one-week of acclimatization, the mice were divided into five groups (*n* = 6): normal control (NC), model (MD), positive control (PC), and ZLE treatment groups (100 and 200 mg/kg/day for LD and HD groups). HX and PO were dissolved in 0.5% CMC-Na solution to establish a HUA mouse model. Except for the NC group, mice in the other groups were received intraperitoneal PO (300 mg/kg/day) and intragastrical HX (300 mg/kg/day) for 21 days (32). The NC group was gavaged and intraperitoneally injected 0.5% CMC-Na of the same volume. One hour later, mice in the PC group and ZLE treatment group were gavaged with 200 μL 5 mg/kg allopurinol and ZLE extracts. The NC group and the MD group were gavaged with 0.5% CMC-Na of the same volume. During which the mice were free to eat and drink water, and the changes in the weight of mice were recorded every week.

#### 2.7.3 Sample collection

On day 21 of treatment, biological specimens were obtained from all experimental groups. Food was withdrawn 12 h prior to euthanasia to minimize metabolic interference. One hour following the last administration, whole blood was drawn and subsequently centrifuged at 3000 rpm for 10 min to separate the serum fraction. The isolated serum was preserved at −80 °C pending biochemical evaluation.

After sacrifice, hepatic tissue and the right kidney were carefully excised, weighed, and stored at −80 °C for subsequent analyses. The left kidney was collected separately and immersed in 4% paraformaldehyde to enable histopathological examination. Organ mass data were documented for comparative assessment.

#### 2.7.4 Biochemical analysis

XOD and LPS in serum and XOD in the liver were detected according to the kit instructions. UA, creatinine (CRE), blood urea nitrogen (BUN), alanine aminotransferase (ALT), aspartate aminotransferase (AST) in serum were measured using a Mindray BS-360S automatic biochemical analyzer (Mindray Medical International Ltd., Shenzhen, China). Caspase-1 activity and the protein levels of Caspase-1, IL-1*β*, and IL-18 in the kidney were determined using assay kits according to the manufacturer’s instructions.

#### 2.7.5 Histological assay of kidney

Kidney tissues were fixed in 4% paraformaldehyde at room temperature for 1 day. Following fixation, tissues underwent dehydration through a graded ethanol series. Dehydrated tissues were then embedded in paraffin blocks. Subsequently, the paraffin blocks were sectioned to obtain thick tissue slices. These sections were finally stained using hematoxylin and eosin (H&E). Stained tissue sections were examined and photographed. Imaging used an OLYMPUS CX43-LP microscope (Olympus Corporation, Tokyo, Japan).

#### 2.7.6 Quantitative real-time PCR analysis

The relative mRNA expression levels of NLRP3, Caspase-1, TNF-*α*, and *β*-actin were analyzed using qRT-PCR. The specific analysis methods and corresponding primer sequences are presented in Appendix A and Table A1.

#### 2.7.7 Analysis of the western blot

To further explain the molecular mechanism of ZLE on renal urate handling, the relative expression levels of GLUT9, URAT1, ABCG2 and OAT1 proteins were analyzed via Western blot. The specific experimental procedures are detailed in Appendix B.

### 2.8 Statistical analysis

Data were presented as mean ± standard deviation. Graphical representations of the results were conducted using ImageJ and GraphPad prism 10.4.2. The untargeted metabolomic analysis was performed using Compound Discoverer 3.3. Differences between groups were considered significant at *p* values less than 0.05. One-way analysis of variance (ANOVA) and Dunnett’s multiple comparison tests were used to analyze statistical differences.

## 3 Results

### 3.1 Residual ethanol determination

Headspace GC-MS analysis detected residual ethanol in the freeze-dried ZLE at a concentration of 0.207% (*w/w*). The residual ethanol levels remain below the limit (0.5%, 5,000 ppm) specified for Class 3 solvents in the International Council for Harmonization (ICH) Q3C Impurities: Residual Solvents guideline. The corresponding ethanol exposure was estimated to be approximately 0.461 ppm in the HepG2 cell model and 0.2–0.4 mg/kg/day in mice.

### 3.2 Main bioactive components in ZLE

Table 1 summarizes the content of major nutrients in ZLE solution. The nitrogenous compounds in ZLE are mainly low-molecular-weight peptides (<10 kDa), with a content of 1.280 ± 0.023 mg/mL. A large number of plant secondary metabolites were detected in ZLE. Among them, the polysaccharides content was as high as 3.809 ± 0.036 mg/mL, the total terpenes content was 2.841 ± 0.039 mg/mL, and the total flavonoid content was 1.098 ± 0.008 mg/mL. These results indicate that ZLE is an extract rich in peptides and polysaccharides.

**Table 1:** The content of proteins, peptides, polysaccharides, terpenes, and flavonoids in ZLE.

Substance	Content (mg/mL)
Peptides (<10 kDa)	1.280 ± 0.023
Proteins (>10 kDa)	0.234 ± 0.021
Polysaccharides	3.809 ± 0.036
Total terpenes	2.841 ± 0.039
Total flavonoids	1.098 ± 0.008

### 3.3 Phytochemical characterization and identification of constituents in ZLE

**Figure 1:**
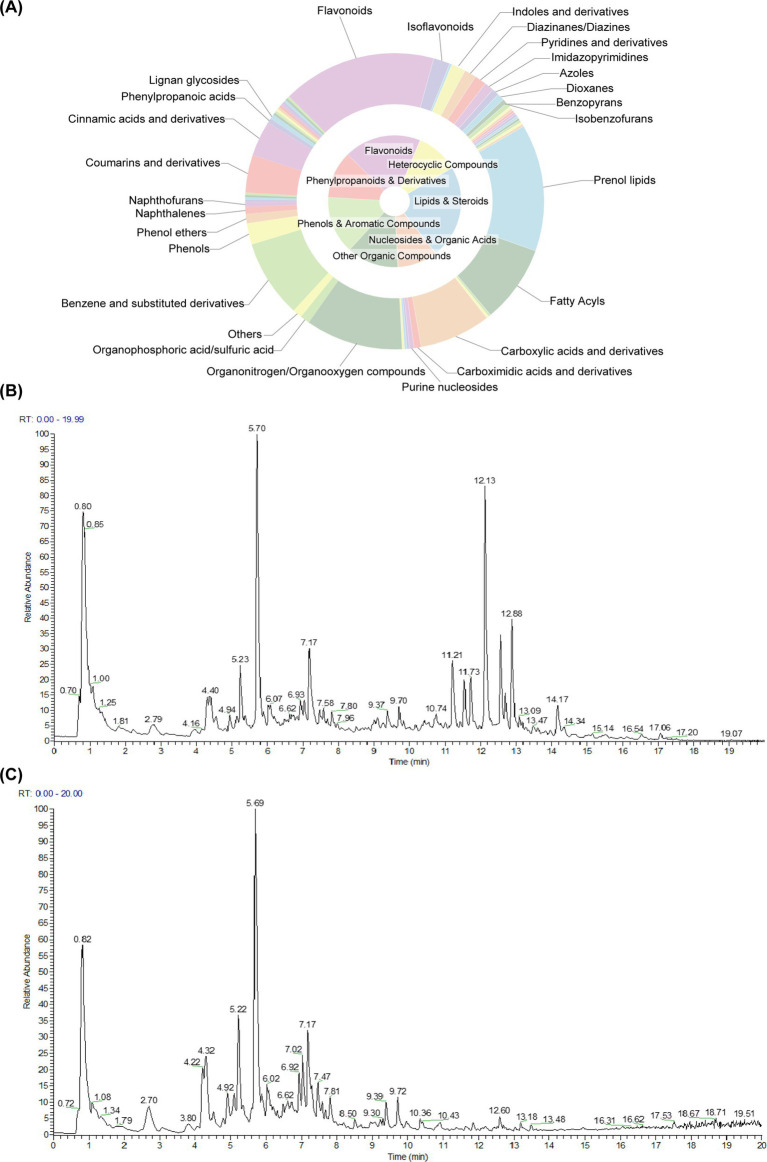
Composition of phytochemicals in ZLE. **(A)** Classification of 389 phytochemicals in ZLE. **(B)** Ion chromatogram in positive ion mode. **(C)** Ion chromatogram in negative ion mode.

A total of 243 and 172 compounds were detected in positive and negative ion modes, respectively. After eliminating 26 duplicated compounds identified in both modes, 389 phytochemicals were obtained. Identification was based on high-resolution MS^2^ data using the Thermo mzCloud and mzVault databases. The classification of these components is presented in a sunburst diagram (Figure 1A). The identified compounds were divided into seven major categories: lipids and steroids, flavonoids, phenols and aromatics, other organic compounds, phenylpropanoids and their derivatives, heterocyclic compounds, and nucleosides and organic acids. Detailed parameters for all components are shown in Supplementary Table S1. The total ion chromatogram (TIC) (Figures 1B,C) shows the rich component composition of ZLE in both positive and negative ion modes.

In pharmacological studies, various classes of phytochemicals have been clearly demonstrated to possess UA-lowering effects, XOD inhibitory activity, or protective effects against renal injury induced by HUA. These include flavonoids (e.g., nobiletin and tangeritin), polyphenols (e.g., chlorogenic acid and 4,5-dicaffeoylquinic acid), phenylpropanoids (e.g., cinnamaldehyde and cinnamic acid), alkaloids (e.g., DL-stachydrine), and iridoid glycosides (e.g., geniposide). To highlight the major bioactive components in ZLE, we analyzed components with a relative abundance > 1%.

In positive ion mode, characteristic components of ZLE include nitrogenous compounds and alkaloids (e.g., DL-stachydrine, choline, and DL-arginine), coumarins (e.g., 4-methylumbelliferone and 7-methoxycoumarin), and various phenylpropanoids (e.g., cinnamaldehyde, cinnamic acid, and 4-methoxycinnamic acid). Notably, polymethoxyflavonoids such as nobiletin and tangeritin were also prominently identified.

In negative ion mode, iridoid glycosides (e.g., geniposide, genipin 1-O-*β*-D-gentiobioside, and shanzhiside) and organic acids (e.g., D-(−)-quinic acid, citric acid, and 4,5-dicaffeoylquinic acid) are dominant. Furthermore, a variety of polyphenolic compounds and flavonoid glycosides, including chlorogenic acid, naringin, and hesperidin, were observed. These results indicate that ZLE is rich in diverse phytochemicals, particularly iridoid glycosides, organic acids, and flavonoids, which likely synergistically contribute to its therapeutic efficacy against HUA.

### 3.4 Anti-hyperuricemic effect of ZLE *in vitro*

**Figure 2:**
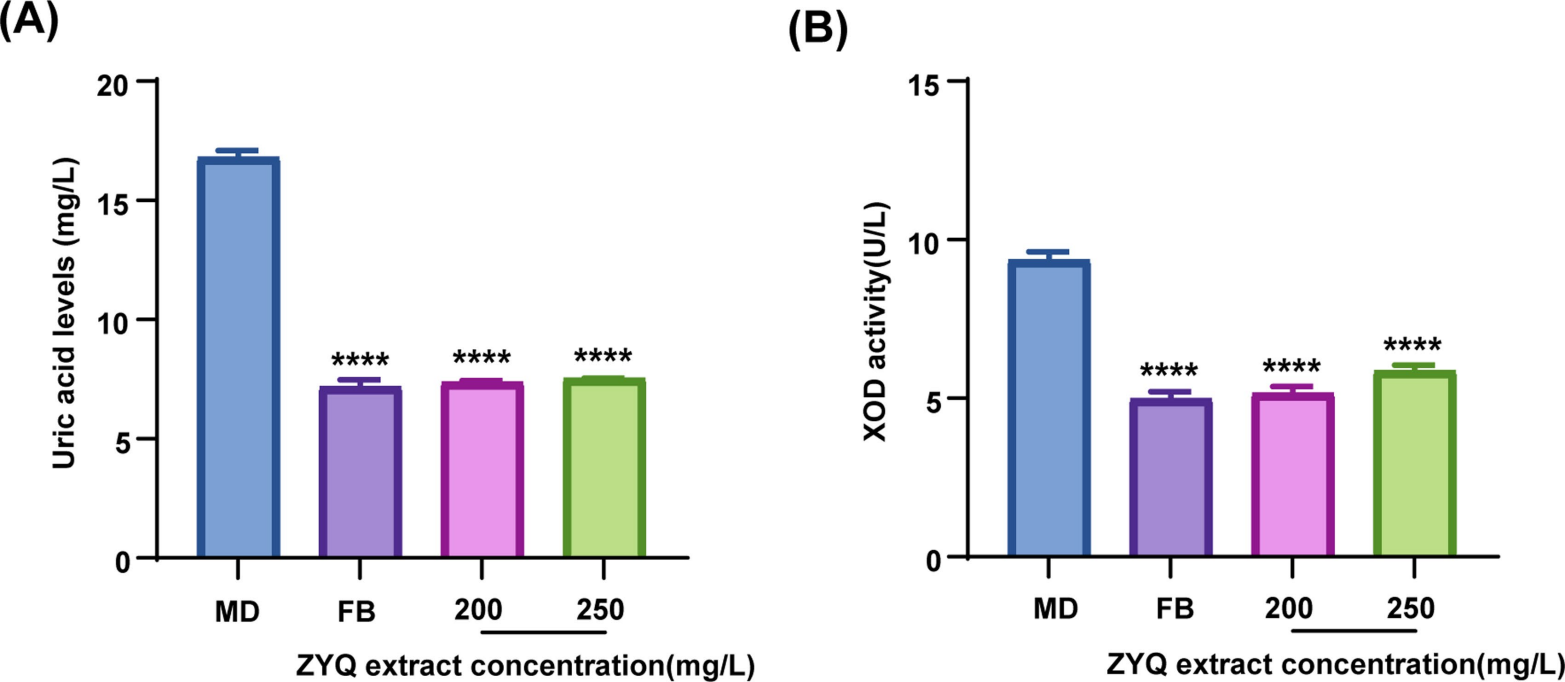
The levels of uric acid and the activity of XOD in cell culture supernatants. **(A)** Uric acid levels. **(B)** XOD activity. Data were expressed as mean ± SEM, **** *p* < 0.0001, compared with the model group. MD, model group; FB, febuxostat group.

Figure 2A shows that compared with the model group, both ZLE and febuxostat intervention significantly reduced the UA level in the supernatant of HepG2 cells (*p* < 0.0001). In addition, both ZLE and febuxostat intervention also significantly reduced the activity of XOD in the cell supernatant (*p* < 0.0001) (Figure 2B). These results indicate that ZLE may have a direct inhibitory effect on UA production at the cellular level by reducing XOD activity, which is comparable to the effect of the positive control drug febuxostat.

### 3.5 ZLE improved systemic health and restored organ indices in HUA mice

**Figure 3:**
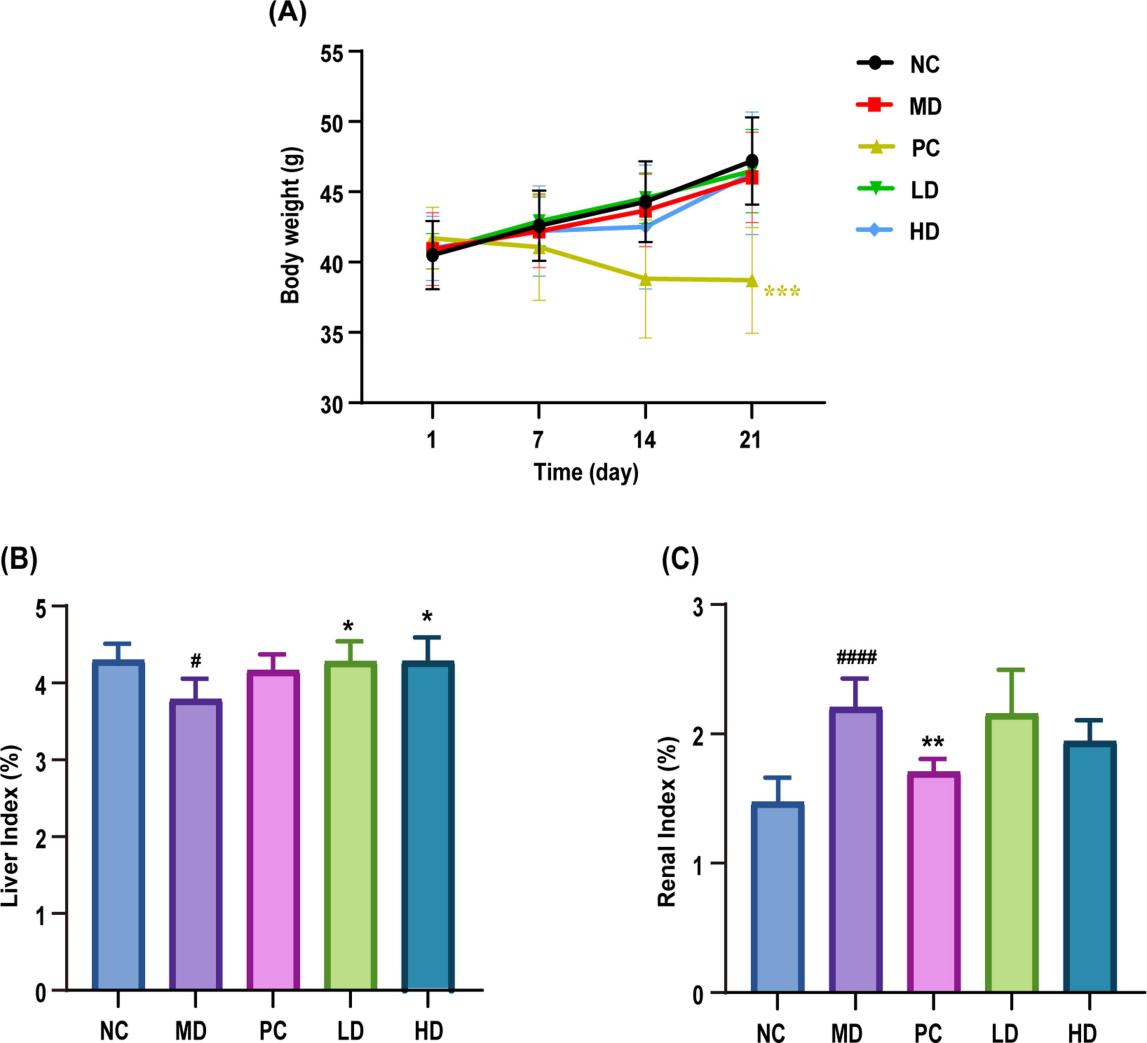
Effects of ZLE on body weight and organ indices in HUA mice. **(A)** Body weight changes throughout the 21-day experiment. **(B)** Liver index. **(C)** Renal index. Data were expressed as mean ± SD, # *p* < 0.05 and #### *p* < 0.0001, compared with the control group; * *p* < 0.05, ** *p* < 0.01, and *** *p* < 0.001, compared with the model group. NC, normal control group; MD, model group; PC, positive control group(allopurinol); LD, low dose ZLE group; HD, high dose ZLE group.

We evaluated the systemic protective effects of ZLE by analyzing body weight, organ indexes, and biochemical markers (Figure 3). After 21 days of intervention, the body weight of the PC group was significantly lower than that of the other groups (*p* < 0.001). The body weight reduction observed in the allopurinol group (Figure 3A). ZLE treatment also counteracts HUA-induced organ stress. Compared with the NC group, the liver index of the MD group was significantly lower (*p* < 0.05). ZLE administration restored the liver index of the model mice to normal levels (Figure 3B). In contrast, the renal index of the MD group showed a significant increase (*p* < 0.0001). The ZLE treatment group had no significant effect on the increased renal index in model mice, while the PC group significantly inhibited the increase in renal index (*p* < 0.01) (Figure 3C). These results indicate that compared with the positive drug allopurinol, ZLE has relatively favorable tolerability and potential liver protection.

### 3.6 Effects of ZLE on serum urate homeostasis and hepatorenal function

#### 3.6.1 Evaluation of serum UA and renal function indicators

**Figure 4:**
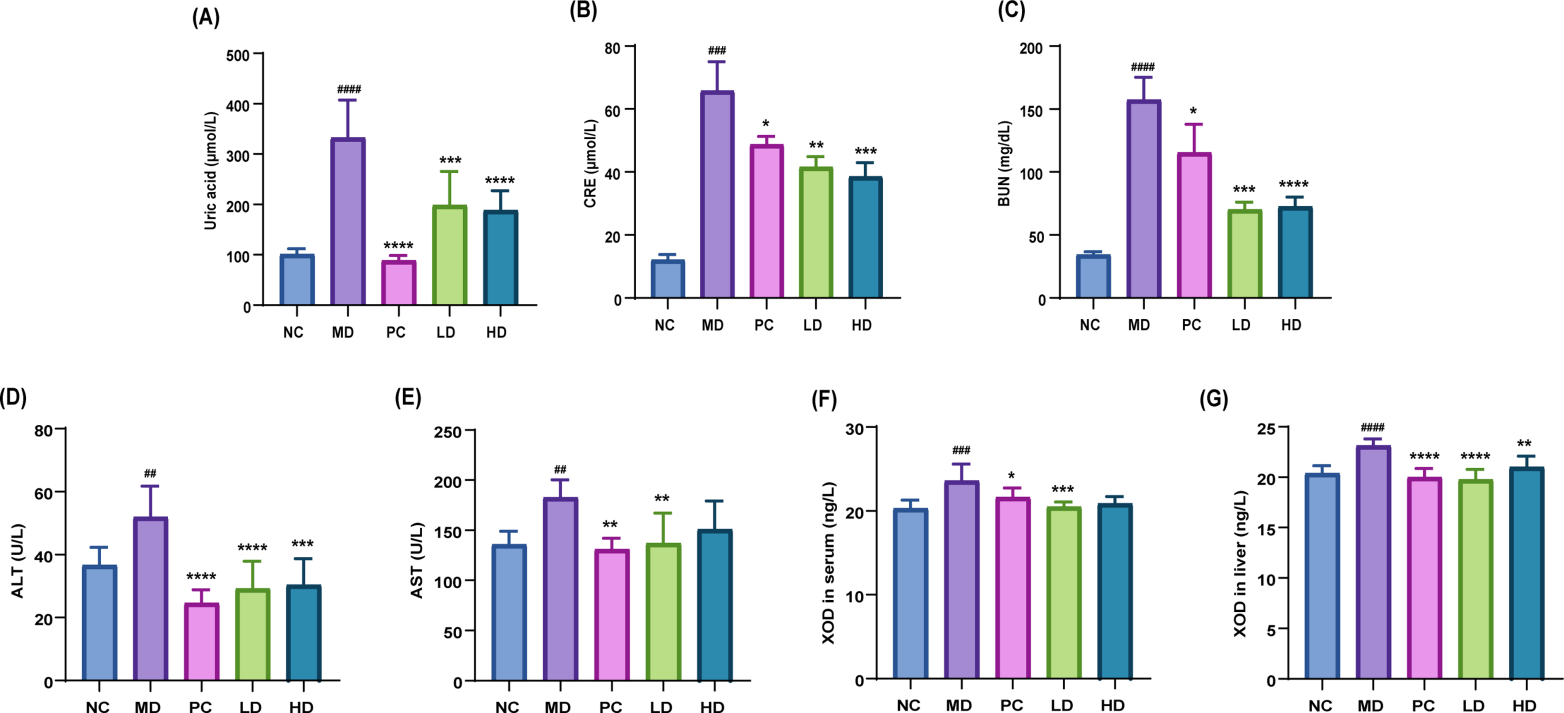
Effects of ZLE on UA levels, liver and kidney injury markers and XOD activity. **(A)** Uric acid in serum. **(B)** CRE in serum. **(C)** BUN in serum. **(D)** ALT in serum. **(E)** AST in serum. **(F)** XOD in serum. **(G)** XOD in liver. Data were expressed as mean ± SEM, ## *p* < 0.01, ### *p* < 0.001 and #### *p* < 0.0001, compared with the control group; * *p* < 0.05, ** *p* < 0.01, *** *p* < 0.001, and **** *p* < 0.0001, compared with the model group. NC, normal control group; MD, model group; PC, positive control group(allopurinol); LD, low dose ZLE group; HD, high dose ZLE group.

HUA resulted in a significant increase in serum UA levels (*p* < 0.0001). ZLE (*p* < 0.001 and *p* < 0.0001) and allopurinol (*p* < 0.0001) treatments significantly reversed this increase (Figure 4A). CRE and BUN are important indicators for evaluating kidney function. HUA caused a significant increase in both CRE and BUN levels in serum (*p* < 0.001 and *p* < 0.0001). ZLE treatment reduced the levels of these indicators more effectively than allopurinol (Figures 4B,C).

#### 3.6.2 Assessment of XOD activity and liver function indicators

Since the liver is the main site for urate biosynthesis and XOD is the key rate-limiting enzyme in the synthesis process, we analyzed XOD activity and serum liver function indicators (Figures 4D–G). HUA resulted in significant increases in serum ALT (*p* < 0.01) and AST (*p* < 0.01) levels. The LD group successfully restored these indicators to normal levels. HUA caused a significant increase in serum XOD (*p* < 0.001) and liver XOD (*p* < 0.0001) activities. Low doses of ZLE effectively inhibited XOD activity at systemic levels (serum, *p* < 0.001) and within metabolic sources (liver, *p* < 0.0001). This dual effect on serum liver function indicators and XOD activity illustrates that ZLE may reduce urate accumulation and attenuate liver damage by inhibiting XOD activity and urate production in the liver.

### 3.7 ZLE inhibited the LPS-mediated NLRP3/Caspase-1 signaling pathway

**Figure 5:**
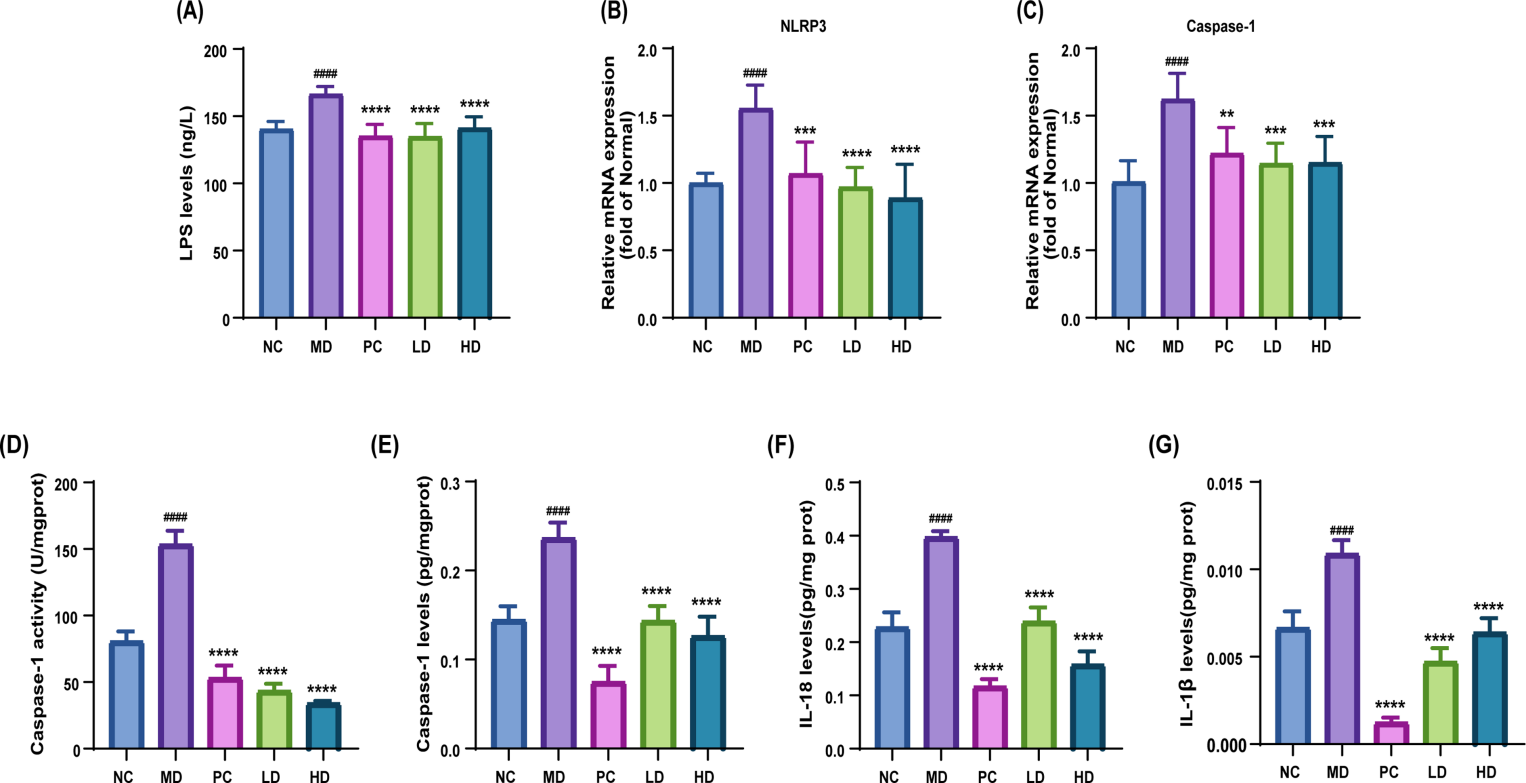
Effects of ZLE on the LPS- mediated NLRP3/Caspase-1 signaling pathway. **(A)** LPS levels. **(B)** Relative mRNA expression of NLRP3. **(C)** Relative mRNA expression of Caspase-1. **(D)** Caspase-1 activity. **(E)** Caspase-1 levels. **(F)** IL-18 levels. **(G)** IL-1*β* levels. Data were expressed as mean ± SEM, #### *p* < 0.0001, compared with the control group; ** *p* < 0.01, *** *p* < 0.001, and **** *p* < 0.0001, compared with the model group. NC, normal control group; MD, model group; PC, positive control group (allopurinol); LD, low dose ZLE group; HD, high dose ZLE group.

In order to study the anti-inflammatory mechanism of ZLE, we detected the levels of serum LPS and the relative expression of renal NLRP3 and Caspase-1 mRNA. ZLE treatment significantly attenuated the HUA-induced increase in serum LPS (*p* < 0.0001), indicating a reduction in systemic inflammatory triggers (Figure 5A). In addition, RT-qPCR results showed that ZLE treatment effectively inhibited the mRNA expression of NLRP3 (*p* < 0.0001) and Caspase-1 (*p* < 0.001) in renal tissue (Figures 5B,C). To further verify inflammasome activation at the protein level, Caspase-1 activity and the protein levels of Caspase-1, IL-1*β* and IL-18 were determined (Figures 5D–G). Compared with the NC group, HUA mice exhibited significantly elevated Caspase-1 activity and increased renal IL-1*β* and IL-18 levels (*p* < 0.0001). ZLE treatment markedly reduced these inflammatory markers, further confirming the suppression of NLRP3 inflammasome activation. These results suggest that ZLE may reduce the renal inflammatory cascade by regulating the LPS-mediated NLRP3/Caspase-1 signaling pathway, thereby protecting the kidney from HUA-induced inflammatory damage.

### 3.8 ZLE regulated renal urate transporters to promote UA excretion

**Figure 6:**
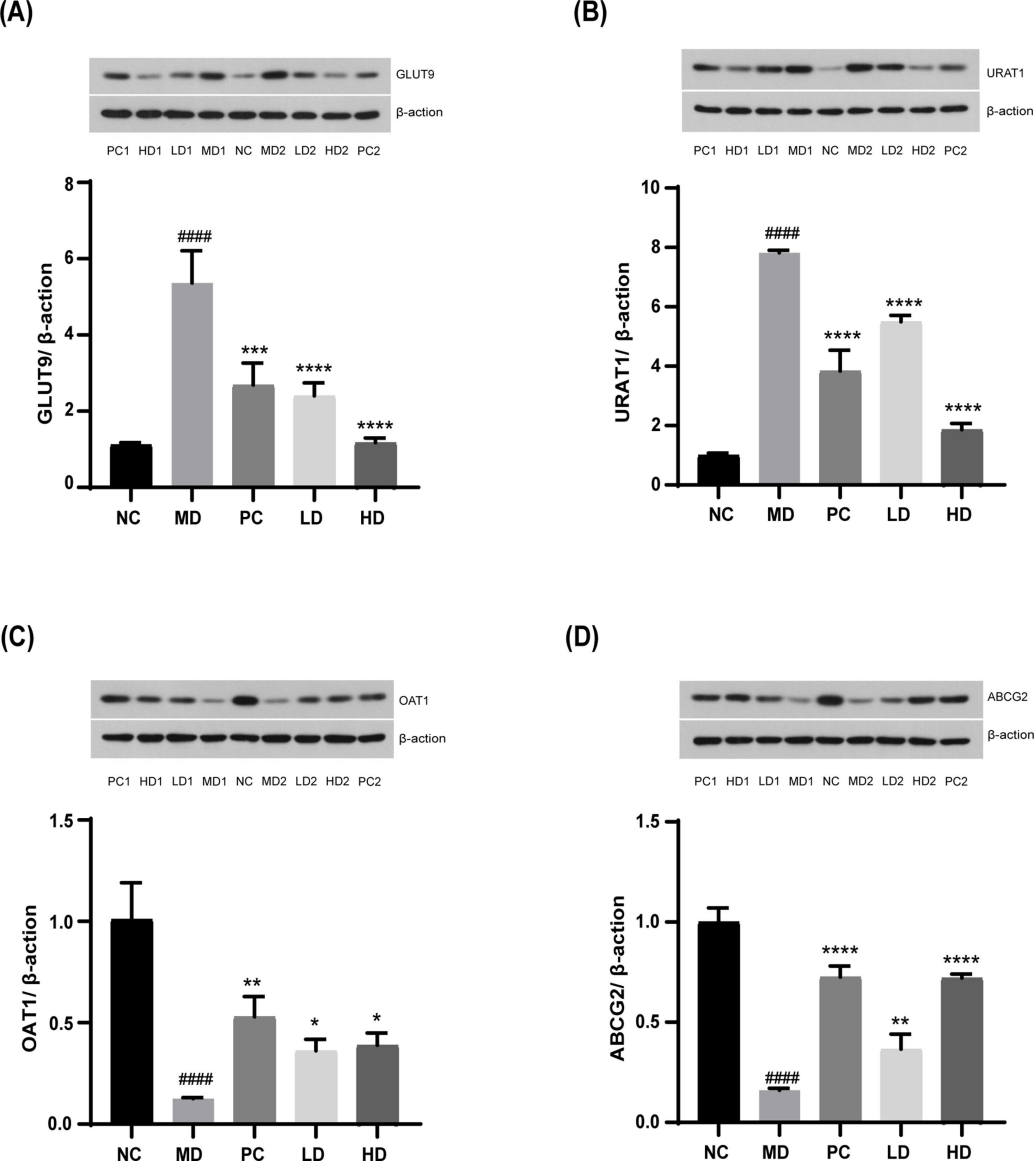
Effect of ZLE intervention on the relative expression of urate transporters. **(A)** GLUT9. **(B)** URAT1. **(C)** OAT1. **(D)** ABCG2. Data were expressed as mean ± SEM, #### *p* < 0.0001, compared with the control group; * *p* < 0.05, ** *p* < 0.01, *** *p* < 0.001, and **** *p* < 0.0001, compared with the model group. NC, normal control group; MD, model group; PC, positive control group(allopurinol); LD, low dose ZLE group; HD, high dose ZLE group.

Western blot results showed (Figures 6A–D) that compared with the normal control group, the protein expression levels of the reabsorption transporters GLUT9 and URAT1 were significantly increased in the model group (*p* < 0.0001). In contrast, the protein expression of secretory transporters OAT1 and ABCG2 was significantly inhibited in model mice (*p* < 0.0001). ZLE treatment effectively reversed these changes in protein expression levels. The HD group showed significant down-regulation of GLUT9 and URAT1 protein expression levels, and the regulatory effect exceeded that of the PC group. In addition, ZLE significantly upregulated the protein expression levels of OAT1 and ABCG2, thereby enhancing the renal UA secretion capacity. Notably, the regulation of URAT1 and ABCG2 exhibited a clear dose-dependent pattern, indicating that enhancement of renal urate handling may be progressively strengthened with increasing ZLE dosage.

### 3.9 ZLE reduced renal pathological damage and local inflammation

**Figure 7:**
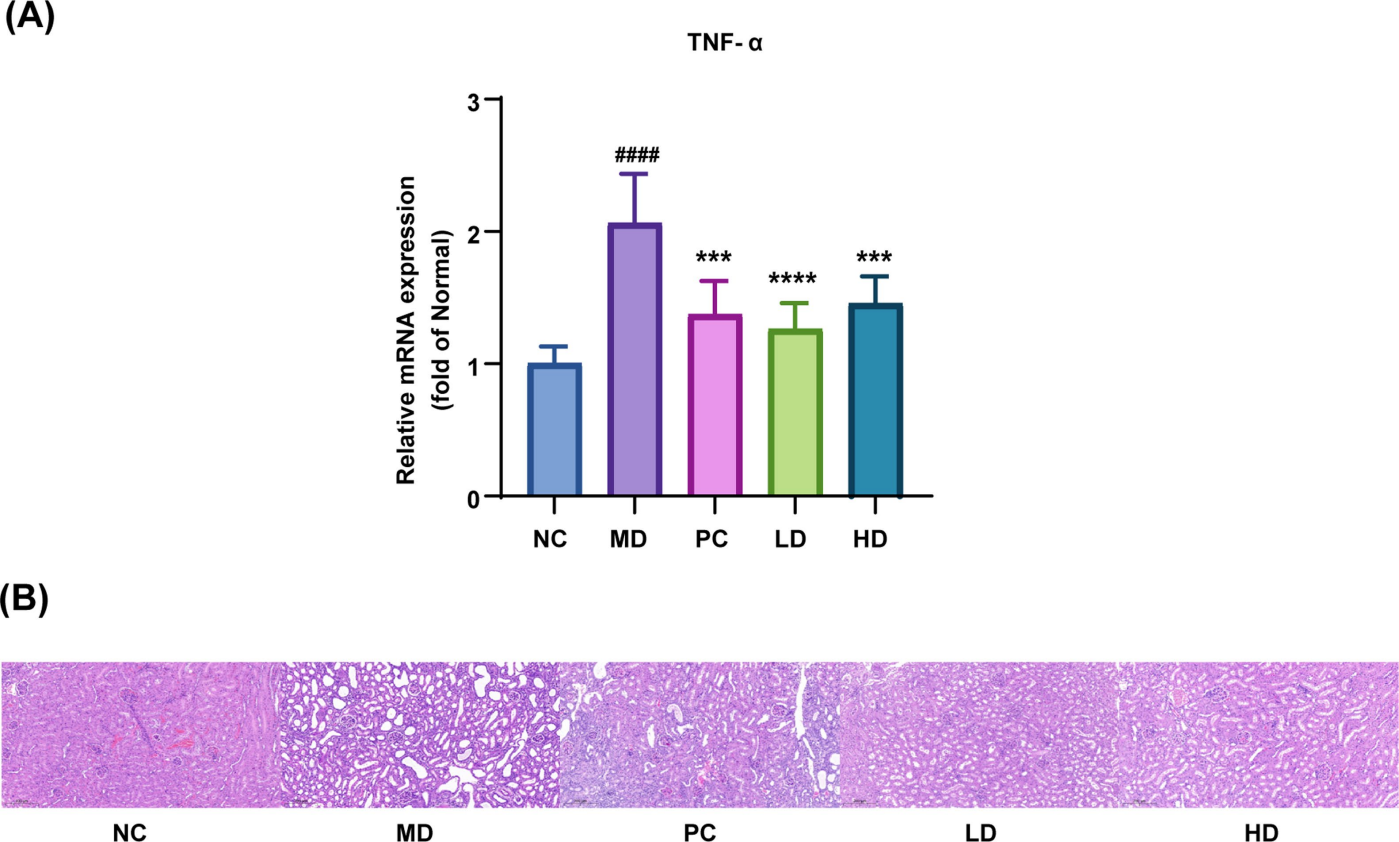
Effect of ZLE intervention on renal pathological damage and local inflammation. **(A)** Relative mRNA expression of TNF-α. **(B)** Staining sections of the kidney. Data were expressed as mean ± SEM, #### *p* < 0.0001, compared with the control group; * *p* < 0.05, ** *p* < 0.01, *** *p* < 0.001, and **** *p* < 0.0001, compared with the model group. NC, normal control group; MD, model group; PC, positive control group(allopurinol); LD, low dose ZLE group; HD, high dose ZLE group.

Restoration of urate homeostasis and molecular signaling was closely associated with maintenance of kidney morphology and reduction of local inflammatory markers (Figures 7A,B). Histological staining showed that the MD group showed obvious tubular swelling, glomerular shrinkage and inflammatory foci. ZLE intervention significantly alleviated these morphological damages, leaving the tubular structure more intact. In addition, ZLE intervention significantly reduced the expression level of pro-inflammatory cytokine TNF-*α* compared with the MD group (*p* < 0.0001). These findings suggest that ZLE can maintain renal structural integrity while suppressing local inflammation.

## 4 Discussion

ZYQ is a functional beverage refined from twelve authentic medicinal herbs, including eight MFH substances such as *Lophatherum gracile* and *Chrysanthemum morifolium*. It has been recorded in the ancient Chinese medical classic *Qimin Yaoshu* (Essential Techniques for the Welfare of the People) and has a consumption history of over 1,000 years. ZYQ is known for its “heat-clearing” and “dampness-removing” properties, which aligns with the nutritional intervention principles of damp-heat syndromes such as gout (11, 33). Phytochemical analysis has shown that ZYQ is rich in bioactive components, mainly including iridoids, flavonoids, terpenes, and phenolic acids (11). Previous biochemical studies of ZYQ have isolated and identified numerous plant compounds from it and revealed its anti-inflammatory properties in cell and animal models, such as inhibiting nitric oxide production and significant hepatoprotective effects against alcohol-induced liver damage (15, 17). Although these studies suggest that certain substances in ZYQ may have potential regulatory effects on UA metabolism, the combination of different plants may have synergistic or antagonistic effects (13, 14). Furthermore, the UA-lowering effects of the functional components in the classic formula ZYQ have not been reported. In this study, ZLE is a freeze-dried extract prepared after ethanol removal and that the study focuses on its phytochemical constituents rather than alcohol itself. Herein, we demonstrated for the first time that ZLE possesses significant anti-hyperuricemic activity. As a dealcoholized extract prepared from a classic formula, ZLE represents a promising source of bioactive compounds for developing functional foods and nutraceuticals against hyperuricemia and associated metabolic disorders.

To further elucidate the bioactive material basis underlying the anti-hyperuricemic effect of ZLE, we conducted a comprehensive chemical profiling of the extract. This analysis revealed that ZLE encompasses a wide array of phytochemicals, with iridoid glycosides, organic acids, and flavonoids being the predominant constituents. Representative compounds such as geniposide, nobiletin, and tangeretin have been reported to exhibit urate-lowering activity through inhibition of XOD and/or modulation of renal urate transporters (20, 34, 35). In addition, cinnamaldehyde and cinnamic acid exhibit dual functionality by competitively inhibiting XOD while simultaneously alleviating HUA-induced gouty arthritis and renal inflammation through suppression of the NLRP3 inflammasome pathway (36). Other constituents, including stachydrine and chlorogenic acid, further contribute to the overall activity, with reported urate-lowering, renoprotective, and antioxidant effects, the latter also reducing XOD activity via competitive inhibition (37, 38). Collectively, this chemically diverse composition provides a coherent and synergistic basis for the multi-target bioactivities observed in the present study (39).

Given that several phytochemicals identified in ZLE have been reported to inhibit XOD activity, we further explored whether suppression of UA production contributed to its anti-hyperuricemic effect. One of the key mechanistic findings was the potent inhibition of XOD by ZLE. As the rate-limiting enzyme in purine metabolism, XOD catalyzes the final step in UA production: the oxidation of HX to xanthine, and then the oxidation of xanthine to UA. Importantly, this catalytic process generates reactive oxygen species (ROS). Therefore, excessive activation of XOD can lead to HUA and associated oxidative tissue damage (3). While allopurinol remains the clinical gold-standard XOD inhibitor, body weight reduction was observed in the allopurinol-treated mice under the present experimental conditions. In contrast, ZLE maintained body weight and organ indices throughout the intervention period, suggesting acceptable tolerability. Consistent results were also obtained in the HepG2 cell model, where ZLE significantly reduced both intracellular UA production and XOD activity.

The kidneys are key organs for maintaining UA homeostasis, performing this function by regulating the delicate balance between reabsorption and secretion. Reabsorption proteins transport UA back into the systemic circulation to maintain necessary physiological levels, while secretory proteins simultaneously pump excess UA into the renal tubules to ensure that net excretion matches the daily metabolic load (40, 41). In HUA mice, we observed upregulation of reabsorption transporters (URAT1, GLUT9) and downregulation of secretory transporters (OAT1, ABCG2). ZLE intervention significantly restored the expression of these proteins. Furthermore, compared with allopurinol, high-dose ZLE exerted a stronger regulatory effect on URAT1 and GLUT9. This enhanced modulation of urate transporters may contribute to the improved renal histopathological outcomes observed in ZLE-treated mice ZLE significantly reversed these alterations and restored a more balanced urate transport profile (4).

In addition to transporter regulation, ZLE also has unique anti-inflammatory advantages. Induction of HUA is accompanied by elevated serum LPS levels, a key indicator of intestinal barrier dysfunction and facilitating the entry of inflammatory ligands into the bloodstream. This systemic increase in LPS levels typically triggers a renal inflammatory cascade. Crucially, ZLE treatment reduced serum LPS levels while downregulating the mRNA expression of TNF-*α*, NLRP3, and Caspase-1. The results provided additional evidence that ZLE suppressed inflammasome activation, as reflected by reduced Caspase-1 activity and decreased levels of the downstream cytokines IL-1*β* and IL-18. These findings suggest that ZLE attenuates LPS-associated renal inflammation through suppression of inflammasome activation (9, 10). Histopathological results confirmed this systemic improvement, with ZLE significantly reducing renal tubular swelling and glomerular atrophy.

Several limitations of the present study should be acknowledged. First, the PO/HX-induced mouse model mainly reflects UA overproduction resulting from uricase inhibition and enhanced purine metabolism, whereas most clinical HUA cases are characterized by impaired renal urate excretion (42, 43). Moreover, systemic inhibition of uricase by PO affects multiple tissues and therefore does not fully recapitulate the pathophysiology of human HUA. Second, although the present study demonstrated the urate-lowering and renoprotective effects of ZLE, the specific phytochemicals responsible for these activities remain to be identified. Future studies combining bioactivity-guided fractionation, transporter-deficient animal models, and long-term dietary intervention approaches are required to further clarify the active constituents and validate the nutritional application potential of ZLE (44).

## 5 Conclusion

This study provides evidence that dealcoholized zhuyeqing liquor extract (ZLE) is a multi-target mechanism involving uric acid (UA) metabolism and inflammation regulation for dietary management hyperuricemia (HUA). ZLE exhibited significant inhibitory effects on xanthine oxidase (XOD) in both cell and animal models, effectively suppressing UA metabolism. More importantly, ZLE restored renal UA homeostasis by regulating key UA transport proteins. In addition to metabolic regulation, ZLE also significantly reduced systemic inflammatory markers such as serum LPS and alleviated renal injury. ZLE was well tolerated under the experimental conditions and did not adversely affect body weight or organ indices. The current study provides a scientific foundation for the development and modernization of dealcoholized bioactive extracts from classic medicinal formulas.

## Data Availability

The original contributions presented in the study are included in the article/Supplementary material, further inquiries can be directed to the corresponding authors.

## Supplementary material

SUPPLEMENTARY TABLE S1 Phytochemical profile of ZLE characterized by UPLC-Q-Exactive HF-MS.

## Appendix

### Appendix A

Kidney tissue (50–100 mg) was homogenized with 1 mL of TRIzol reagent, and total RNA was extracted. Total RNA was reverse-transcribed into cDNA using SuperScript III (Invitrogen, United States). Target gene transcripts were then quantified via RT-qPCR using the SYBR Premix Ex Taq™ Kit (Takara, Japan) on a CFX Connect System. The amplification program consisted of an initial 30 s denaturation at 95°C, followed by 40–45 cycles of 95°C for 10 s and 60°C for 30 s, with a final melt curve analysis (65–95°C). Relative expression levels were normalized and calculated using the 2^–ΔΔCT^ method (45). Specific primer sequences are detailed in Table A1.

**Table A1:** The primer sequences of related genes.

Genes	Forward (5’-3’)	Reverse (5’-3’)
NLRP3	TCCACAATTCTGACCCACAA	ACCTCACAGAGGGTCACCAC
Caspase-1	GGGCCCCAGGCAAGCCAAATC	AGGGCAAGACGTGTACGAGTGGT
TNF-α	AAAGCATGATCCGGGACGTG	CAAAGTGCAGCAGGCAGAAG
*β*-action	AGAGGGAAATCGTGCGTGAC	CAATAGTGATGACCTGGCCGT

### Appendix B

Total protein was extracted from the stored kidney tissues using radio immunoprecipitation assay (RIPA) lysis buffer supplemented with protease and phosphatase inhibitors. The protein concentrations were quantified using a Pierce™ BCA Protein Assay Kit (Thermo Fisher Scientific, Waltham, MA, United States) according to the manufacturer’s instructions.

Equal amounts of protein (30 μg) were denatured and separated by 10% sodium dodecyl sulfate-polyacrylamide gel electrophoresis (SDS-PAGE) gel electrophoresis. The separated proteins were then transferred to a polyvinylidene fluoride (PVDF) membrane. The membrane was blocked at room temperature for 1 hour using 5% skim milk powder dissolved in tris-buffered saline with tween-20 (TBST) buffer. After blocking, the membrane was incubated overnight at 4°C with primary antibodies. The antibodies included GLUT9, URAT1, ABCG2, OAT1, and *β*-actin; all antibodies were diluted 1:5000. After washing three times with TBST, the membranes were incubated with the corresponding horseradish peroxidase (HRP)-conjugated secondary antibodies for 1.5 h. Protein bands were visualized using an enhanced chemiluminescence (ECL) detection system. Densitometric analysis was performed using Gel-Pro Analyzer version 4.0 software (Media Cybernetics, MD, United States), and the relative expression levels of target proteins were normalized to the gray value of *β*-actin.

## Glossary

HUA: Hyperuricemia
ZLE: Dealcoholized zhuyeqing liquor extract
MFH: Medicine and food homology
UA: Uric acid
XOD: Xanthine oxidase
URAT1: Urate anion transporter 1
GLUT9: Glucose transporter type 9
OAT1: Organic anion transporter 1
ABCG2: ATP-binding cassette subfamily G member 2
LPS: Lipopolysaccharide
NLRP3: NOD-like receptor thermal protein domain associated protein 3
ZYQ: Zhuyeqing liquor
TCM: Traditional Chinese Medicine
Caspase-1: Cysteine-aspartic acid protease 1
HX: Hypoxanthine
CMC: Sodium carboxymethyl cellulose
MTT: 3-(4,5-Dimethylthiazol-2-yl)-2,5-diphenyltetrazolium bromide
DMSO: Dimethyl sulfoxide
PO: Potassium oxazinate
DMEM: Dulbecco’s modified eagle’s medium
FBS: Fetal bovine serum
SPF: Specific-pathogen-free
CRE: Creatinine
BUN: Blood urea nitrogen
ALT: Alanine aminotransferase
AST: aspartate aminotransferase
HRP: Horseradish peroxidase
ECL: Enhanced chemiluminescence
ANOVA: One-way analysis of variance
TIC: Total ion chromatograms
ROS: Reactive oxygen species
HCD: Higher collisional dissociation
DDA: Data dependent acquisition
MWCO: Molecular weight cut off
UHPLC: Ultra-high performance liquid chromatography
RIPA: Radio immunoprecipitation assay
ESI: Electrospray ionization
SDS-PAGE: Sodium dodecyl sulfate-polyacrylamide gel electrophoresis
PVDF: Polyvinylidene fluoride
TBST: Tris-buffered saline with tween-20
SIM: Selected ion monitoring
EI: Electron ionization
GC-MS: Gas chromatograph-mass spectrometric
UPLC-Q-Exactive HF-MS: Ultra-performance liquid chromatography coupled with quadrupole-exactive HF mass spectrometry
